# The Chinese version of the Oral Health Impact Profile-14 (OHIP-14) questionnaire among college students: factor structure and measurement invariance across genders

**DOI:** 10.1186/s12903-022-02441-6

**Published:** 2022-09-17

**Authors:** Yao Feng, Jing-Jie Lu, Ze-Yue Ouyang, Lan-Xin Xue, Tan Li, Yun Chen, Zheng-Rong Gao, Shao-Hui Zhang, Jie Zhao, Ya-Qiong Zhao, Qin Ye, Jing Hu, Yun-Zhi Feng, Yue Guo

**Affiliations:** 1 Department of Stomatology, The Second Xiangya Hospital of Central South University, Changsha, 410011 Hunan China; 2 Medical Psychological Center, The Second Xiangya Hospital of Central South University, Changsha, 410011 Hunan China; 3 Xiangya Stomatological Hospital, Central South University, Changsha, 410000 Hunan China; 4 Department of Stomatology, Xiangyang central hospital, Xiangyang, China

**Keywords:** The Oral Health Impact Profile-14 (OHIP-14), China, Oral Health-related Quality of life (OHRQoL), Factor structure, Reliability, Measurement invariance, College students

## Abstract

**Background:**

The Oral Health-related Quality of Life (OHRQoL) is a multi-dimensional concept commonly used to examine the impact of health status on quality of life, and the Oral Health Impact Profile-14 (OHIP-14) questionnaire is a good self-assessment tool. This study was designed to investigate the factor structure of the OHIP-14 scale Chinese version, measurement invariance and latent mean differences across genders among college students.

**Methods:**

The online survey was completed by 919 college students. This study used confirmatory factor analysis (CFA) to check the structural models of the OHIP-14 scale, The correlation of each item with the scale total score could test homogeneity, and Cronbach’s alpha (Cronbach’s α) could evaluate internal consistency. Multi-group CFA was used to explore whether the Chinese version of the OHIP-14 scale was used in male and female populations for measurement consistency. T-test compared scores between men and women. Regression analyses were used to evaluate the relationship between age, gender, education, subject, and the score on the OHIP-14 scale.

**Results:**

We found that the 7-factor structure had the best fit index in the sample. According to Cronbach’s α, the overall score of OHIP was 0.958, and Cronbach’s α for 7 factors was: functional limitation was 0.800, physical pain was 0.854, psychological discomfort was 0.902, physical disability was 0.850, psychological disability was 0.768, social disability was 0.862, social handicap was 0.819 and the test–retest reliability interval was 0.723. Multi-group confirmatory factor analysis supported residual measurement invariance across gender. T-test for scores showed that females scored higher significantly than men as did the overall score, in terms of physical pain (*p*<0.001), physical disability (*p*<0.001), and psychological disability (*p*<0.001).

**Conclusions:**

This study found the OHIP-14 Chinese version to be a good tool for assessing the college students' OHRQoL in China, allowing people to conduct self-assessments.

**Supplementary Information:**

The online version contains supplementary material available at 10.1186/s12903-022-02441-6.

## Background

The Oral Health-related Quality of Life (OHRQoL) is an essential part of health and wellbeing that aims to assess the impact of oral health on aspects of personal and social life. OHRQoL is also one of the ten standards of human health formulated by the World Health Organization. According to the Global Burden of Disease Study 2017, it was estimated that nearly 3.5 billion people worldwide suffer from oral disease [1]. To promote health, develop health policies and plan healthcare services, we need to have a clear picture of the health status as well as the trends among a given population [2]. Relevant studies have shown that periodontitis, dental trauma, dental caries, temporomandibular joint (TMJ), and other oral diseases and risk factors have different effects on men and women [3, 4]. Periodontitis and dental trauma were higher in men [4, 5], whereas dental caries and TMJ pain were more prevalent in women [6, 7]. Therefore, gender difference would be an important factor affecting OHRQoL, and it is necessary to explore the role of gender factors in oral health.

OHRQoL is defined as a multidimensional construct encompassing physical, social, and psychological areas [8]. To measure OHRQoL, specific instruments were used, such as the Geriatric Oral Health Assessment Index (GOHAI) [9], Oral Impacts on Daily Performances (OIDP) [10], and the Oral Health Impact Profile (OHIP) [11]. Different scales may focus on different dimensions and populations, and the number of items on the scales would vary. OHIP is the most widely used scale, and its short form OHIP-14 was widely accepted because of its short version and good feasibility in clinical application [12]. The OHIP-14 has been widely applied in different samples and contexts, for example, in the elderly [13], the general population (non-patient) [14], and edentulous subjects patients [15]. However, previous studies showed different evidence for factor structure. Four domains of the structure were found in Chinese adults, including physical disability, psychological disability, social disability, and physical pain [16]. A set of 3 underlying factors, like functional limitation, pain discomfort, and psychosocial impacts, were confirmed by Javier Montero et al. [17]. Recent studies have confirmed the adequacy of the original factorial structure, the 7-factor structures of OHIP-14 among university students [18]. It illustrated the need to explore appropriate structural factors for the OHIP-14 scale to make sure that it is utilized as an important tool for assessing OHRQoL.

In addition, the OHIP-14 scale has been used to evaluate the effect of gender on OHRQoL, but the results have been surprisingly inconsistent. In a cross-sectional study of Turkish dental patients using the OHIP-14 scale, it was found that the OHRQoL of male dental patients was higher than that of female patients [19]. In a study investigating the gender comparison of OHRQoL and its relationship with oral health parameters in the elderly in Wroclaw, southwestern Poland, no gender differences in oral health parameters were found [20]. Considering the contradictory results, there was a need to determine the invariance of the measurement of  the OHIP-14 in males and females and to determine whether the differences between the groups shown by the study results were actual differences or due to measurements. Measurement invariance referred to the measurement model equivalence of the relationship between observed variables and potential variables in different populations or between different populations in the same population. Before testing means differences across groups, it was essential to assess the invariance of the construct [21]. There are few studies on the gender invariance test of the OHIP-14 in China, so it is necessary to investigate the psychological characteristics of OHRQoL in both male and female populations to ensure that its function is the same as the original scale.

From 1892 to 2015, China conducted four nationwide oral epidemiological surveys, which were relevant research for the age categories of 3–5 years old, 12–15 years old, 35–44 years old, and 65–74 years old, but the age of the survey population has not yet included college students [22, 23]. College students were in a period of growth and development, and some studies have found that the unhealthy eating habits of college students were more common. For various reasons, they would ignore the quality of breakfast or have irregular meals, insufficient intake of dairy products, excessive intake of high-sugar beverages, often eat Western-style fast food, and snacks that were mostly high-sugar and high-salt foods [24]. Because of these reasons, it was more likely to lead to oral health problems, which in turn affected the quality of life-related to oral health [25, 26]. Changes in college students’ oral health could affect their physical condition. Moreover, it could also affect a person's appearance, self-esteem, psychosocial functioning, and quality of life. Studies have shown that oral diseases could cause psychological burdens [27]. The quality of life in this age group is critical. Therefore, monitoring, evaluating, and taking measures to improve the OHRQoL level of college students is of great significance.

College students, as part of the society who are about to enter the society, are facing pressures from interpersonal, academic, and employment aspects. If their oral health status is in a bad condition, it will adversely affect their mental health over time. Therefore, the self-assessment of OHRQoL is of great practical significance. OHIP-14 scale is often used in psychology and behaviors to measure OHRQoL data. Psychometrics is a scientific field that focuses on developing tools for assessing, measuring, and connecting observable phenomena. However, it is a pity that so far there are few studies examining the psychometric performance of the OHIP-14 among Chinese college students. For this reason, this study took Chinese college students as participants to assess the appropriateness and fidelity of the OHIP-14 scale in measuring OHRQoL. We aimed to explore the good fit model structure and test the reliability of the OHIP-14 Chinese version, especially an in-depth analysis of its invariance in gender, in order to provide scientific evidence for reference for the application of the OHIP-14 scale in China.

## Methods

In this study, confirmatory factor analysis (CFA), item analysis and reliability, multi-group CFA, and T-test were used to analyze the collected data. CFA can check the structural models of the OHIP-14 scale across the Chinese college students, that is, whether the OHIP-14 scale is good, whether the scale items are good, and whether the data collected reflects the expected results, is actually a validity test. The correlation of each item with the scale total score can test homogeneity, and Cronbach’ alpha (Cronbach’s α) can evaluate internal consistency, which actually is a internal consistency test. Regression analyses were performed on scores of OHIP-14. Age, gender, education level, and subjects were entered as independent variables. Since gender is often used as a potential factor affecting the measurement effect of measurement tools, multi-group CFA in this paperstudy can be used to explore whether the Chinese version of the OHIP-14 scale is used in male and female populations for measurement consistency. Because the sample size of this study is the same and the statistical significance is the same, the T-test can be used to compare scores between men and women. Such testing procedures can reflect whether the questionnaire accurately assesses what it measures and is successfully applied to the target population.

### Participants

In this study, a total of 938 college students were invited by convenience sampling from October to December of 2021, including 212 from Central South University, 302 from Hunan University of Technology and Business, and 424 from Changsha Aviation Vocational and Technical College. 19 of them were excluded due to the lack of data in the questionnaire. Therefore, the final sample of college students enrolled in this study was 919. All participants were generally healthy university students without oral conditions such as oral cancers, congenital craniofacial deformities, craniofacial trauma, etc. We also excluded students who were suffering from systemic disease and mental sickness. Table 1 and Additional file 1: Table S1 presents the general characteristics of the samples. The study was conducted following the ethical principles of the World Medical Association Declaration of Helsinki and was approved by the Human Experiment and Ethics Committee, the Second Xiangya Hospital of Central South University (reference number: KQ2019FY01). Reporting of data was based on the Strengthening the Reporting of Observational Studies in Epidemiology (STROBE) guidelines [28].

**Table 1:** Demographic and medical data of the university subjects (n = 919)

Variables	All subjects (n = 919)	
	Mean/N	%
Years of age(SD)	20.18 (2.472)	
Gender		
Male	536	58.3
Female	383	41.7
Educational level (candidate)		
Bachelor	790	85.9
Master	109	11.9
Doctor	20	2.2
Subject		
Arts	233	25.4
Science	299	32.5
Medical science	135	14.7
Others	252	27.4

In addition, after a one-month interval between separate administrations of the questionnaire, around 10% of the sample (n = 104) were invited to retest OHIP-14 to evaluate its stability.

### Instruments

The OHIP-14, a specific instrument, is the most widely used both by researchers and clinicians to measure OHRQoL [29]. The OHIP-14 comprises 14 items that describe 7 dimensions: functional limitation, physical pain, psychological discomfort, physical disability, psychological disability, social disability, and social handicap. The format for the questions is “In the past month, have you had … because of problems with your teeth, mouth, or dentures?” Items are scored on a 5-point Likert scale ranging from 0 (“never”) to 4 (“very often”). Summary scores (OHIP-14) were derived by summing the response codes across all 14 items (possible range 0–52), with lower the OHIP-14 scores indicating better OHRQoL. Based on the Guidelines for cross-cultural adaptation of health-related quality of life measures, the OHIP-14 scale has been translated into Chinese version through the following five steps: initial translation, synthesis of the translations, back translation, expert committee, a test of the prefinal version [16, 30, 31]. Above studies showed that the OHIP-14 Chinese version has strong evidence of good psychometric performance, which demonstrated good reliability (Cronbach’s α = 0.93) and validity (corrected item-total correlation ranged from 0.53 to 0.71) [16].Also, because of its widespread use and concise and easy-to-understand content, there is no annoying burden when people fill out the OHIP-14 scale [32–35].

### Data analysis

Confirmatory factor analysis (CFA) and measurement invariance analyses were performed with Mplus 8.3 with the robust weighted least squares with mean and variance adjustment estimator (WLSMV). All other analyses were conducted with the IBM SPSS version 26. Initially, the psychometric sensitivity of the items was verified through descriptive statistics (mean, median, standard deviation (SD), minimum and maximum values) of the answers given by all subjects. Univariate normality assumptions (for each item of OHIP-14) were tested by computing Skewness and Kurtosis, and all the items lay within the recommended range of skew and kurtosis coefficients, which should not be above 3 and 10, respectively [36].

#### Confirmatory factor analysis

The OHIP-14 has been widely applied in different samples and contexts. Given the different proposals for factor structures for OHIP-14 [16, 18], We used the CFA method to evaluate the fit of different factorial models of the OHIP-14 (3-factor model, 4-factor model, and 7-factor model) for Chinese University Students before testing measurement invariance [37]. Model fit was estimated using the χ^2^ statistic, the comparative fit index (CFI), the Tucker–Lewis index (TLI), standardized root mean square residual (SRMR), and the root means the square error of approximation (RMSEA). Following Hu and Bentler [38], we considered the fit of the factorial model to the data was considered adequate when CFI and TLI ≥ 0.95. In addition, SRMR < 0.05 and RMSEA ≤ 0.08 were considered to indicate a satisfactory fit.

#### Item analysis and reliability

We assigned the correlation of each item with the scale total score to test homogeneity, and the scores above 0.3 were seen as acceptable according to Nunnally and Bernstein [39]. Cronbach’s α was used to evaluate internal consistency, and if the coefficient was above 0.600, it was seen as acceptable for the total score or dimension scores, respectively [40]. Test–retest reliability was estimated through the intraclass correlation coefficient (ICC) [41].

#### Factorial invariance

To determine whether the measurement model could be equivalent across genders, multiple-group CFA was conducted to assess the measurement invariance of the OHIP-14 [42]. Invariance procedures were followed: (1) We tested the best-fit model from CFA in men and women separately. (2) Configural invariance model (M1), metric (weak) invariance model (M2), scalar (strong) invariance model (M3), and residual invariance model (M4) should be constrained to be equal across genders, which meant each model was compared with its preceding model to study whether the model fit deteriorated significantly. A non-significant Δχ^2^ difference test (Δχ^2^ test) was considered as evidence of invariance. However, the Δχ^2^ test sensitivity to sample size, we also relied on models’ differences in CFI (ΔCFI), where values < 0.01 of ΔCFI [43, 44]. Also, a comparison of participants in female and male is shown in Table 5. T-test was applied to compare the statistically significant of each item between the female and male groups.

## Results

### Participants’ background

Table 1 shows the basic demographic characteristics of the university subjects. The average age of all subjects was 20.18 ± 2.472. 58.3% of participants were men, 41.7% were women and 85.9% of them were bachelors. 32.5% of students majored in Science discipline, while 25.4% and 14.7% of students majored in Arts and Medical science respectively. Results of regression analysis with age, gender, education, and subject as outcomes are presented in Additional file 1: Table S1. Age (0.429 [95% CI 0.161–0.197], *p* = 0.002), gender (−1.636 [95% CI −2.858 to −0.413], *p* = 0.009), and arts subjects (3.165 [95% CI 0.570–4.759], *p* = 0.000) were associated with OHIP-14 scores.

### Descriptive statistics of study subjects

The prevalence of impact on OHRQoL was 31.84% and the mean OHIP-14 score was 5.751 ± 8.7388 (range 0-52). The highest mean scores were observed for the dimension of physical pain, including Item 3 (0.594 ± 0.8964) and Item 4 (0.526 ± 0.8461), and followed by physical disability, including Item 7 (0.594 ± 0.8642) and Item 8 (0.455 ± 0.8151). The psychological discomfort dimension, covering Item 5 (0.298 ± 0.7382) and Item 6 (0.311 ± 0.7417), was the lowest mean score. In addition, the distribution of each item was close to normality (Table 2).

**Table 2:** Descriptive statistics of the responses given to the items of the OHIP-14 by the participants

Item	Mean	SD	Min	Max	Skewness	Kurtosis
It1	0.387	0.7642	0	4	2.328	5.607
It2	0.357	0.7372	0	4	2.365	5.767
It3	0.594	0.8964	0	4	1.398	1.149
It4	0.526	0.8461	0	4	1.564	1.775
It5	0.298	0.7382	0	4	2.768	7.501
It6	0.311	0.7417	0	4	2.657	7.005
It7	0.594	0.8642	0	4	1.291	0.775
It8	0.455	0.8151	0	4	1.942	3.556
It9	0.452	0.7918	0	4	1.848	3.087
It10	0.435	0.7975	0	4	1.962	3.619
It11	0.321	0.6894	0	4	2.400	5.953
It12	0.309	0.6734	0	4	2.414	5.816
It13	0.428	0.8064	0	4	2.097	4.303
It14	0.284	0.6542	0	4	2.672	7.745
Total	5.751	8.7388	0	52	1.840	3.495

### Confirmatory factor analysis

The results of the CFA of the assumed unifactorial factor model, 3-factor model, 4-factor model, and 7-factor model are shown in Table 3. Results suggested that 7-factor model was excellently adequate fit to the University subjects, and the results were as follows: χ^2^ = 213.458, degrees of freedom = 56, *p* < 0.001; CFI = 0.996, TLI = 0.993, SRMR = 0.013 and RMSEA = 0.055 (0.048–0.063).

**Table 3:** The fit of factorial models of the OHIP-14 in University subjects

Model	χ^2^ (df), *p*	CFI	TLI	SRMR	RMSEA (90%CI)
Unifactorial	888.532 (77) *p* < 0.001	0.979	0.975	0.033	0.107 (0.101–0.113)
3 Factors–1st Order	661.273 (74) *p* < 0.001	0.985	0.981	0.026	0.093 (0.086–0.099)
4 Factors–1st Order	609.915 (71) *p* < 0.001	0.986	0.982	0.025	0.091 (0.084–0.098)
7 Factors–1st Order	213.458 (56) *p* < 0.001	0.996	0.993	0.013	0.055 (0.048–0.063)

*χ*^*2*^ chi-square, *df* degree of freedom, *CFI* comparative fit index, *TLI* Tucker–Lewis index, *SRMR* standardized root mean square residual, *RMSEA* root-mean-square error of approximation, *CI* confidence interval

### Item analysis and reliability

To test the homogeneity of the scale, item-total correlations were used. All of the correlations above the recommended cut-off of 0.300, ranged from 0.759 to 0.862. Correlations between each dimension of the OHIP and the total score ranged from 0.811 to 0.916 (*p* < 0.001). The inter-correlations between factors ranged from 0.593 to 0.864 (*p* < 0.001).

For this coefficient of OHIP total score was 0.958, and the Cronbach’s α of 7 factors were as follows: functional limitation 0.800, physical pain 0.854, psychological discomfort 0.902, physical disability 0.850, psychological disability 0.768, social disability 0.862, social handicap 0.819. They were seen as acceptable for the total score or dimension scores.

The test–retest reliability interval was assessed by intra-class correlation coefficients (ICC) (ICC = 0.723).

### Measurement invariance

The 7-factor model had adequate fit in both the male group and female group. The results for measurement invariance across genders are displayed in Table 4. The 7-factor model configural invariance model fitted the data very well (RMSEA = 0.052 [90% CI 0.043–0.060], CFI = 0.997). A constrained metric invariance model showed an acceptable fit (RMSEA = 0.046 [90% CI 0.037–0.055], CFI = 0.998). Given this support, we proceeded to test for scalar invariance. The scalar invariance model (M3) fitted the data soundly well (RMSEA = 0.035 [90% CI 0.026–0.043], CFI = 0.998). Lastly, the scalar invariance was compared with the residual invariance, which suggested that invariance remained stable with each subsequent model constraint (RMSEA = 0.041 [90% CI 0.033–0.048] CFI = 0.997).

**Table 4:** Measurement invariance model across genders fitting indices and comparison

Model	χ^2^(df),* p*	CFI	TLI	SRMR	RMSEA (90%CI)	Model comparison	Δχ^2^ (Δdf)	ΔCFI
Male (n = 536)	132.097 (56), *p* < 0.001	0.998	0.996	0.013	0.050 (0.039–0.062)			
Female (n = 383)	118.738 (56), *p* < 0.001	0.995	0.992	0.018	0.054 (0.041–0.068)			
Model 1	250.225 (112), *p* < 0.001	0.997	0.995	0.015	0.052 (0.043–0.060)			
Model 2	234.403 (119), *p* < 0.001	0.998	0.996	0.015	0.046 (0.037–0.055)	2 versus 1	4.793 (7), *p* = 0.685	0.001
Model 3	239.616 (154), *p* < 0.001	0.998	0.998	0.016	0.035 (0.026–00.043)	3 versus 2	34.716 (35), *p* = 0.452	0
Model 4	297.202 (168), *p* < 0.001	0.997	0.997	0.021	0.041 (0.033–0.048)	4 versus 3	49.931 (14), *p* < 0.001	− 0.001

Model 1 = configural invariance; Model 2 = metric invariance; Model 3 = scalar invariance; Model 4 =residual invariance

*χ*^*2*^ chi-square, *df* degree of freedom, *CFI* comparative fit index, *TLI* Tucker–Lewis index, *SRMR* standardized root mean square residual, *RMSEA* root-mean-square error of approximation, *CI* confidence interval, Δ change in the parameter

T-test for scores of OHIP between male and female groups is shown in Table 5. Female [7.308 (8.7510)] generally had higher overall scores than males [4.638 (8.5662)]. In addition, females scored higher significantly than men in terms of Physical pain (t = 5.837, *p *< 0.001 ), Physical disability (t = 4.898, *p* < 0.001), and Psychological disability (t = 4.097,* p* < 0.001) in turn.

**Table 5:** T test for scores of the OHIP-14 between male and female groups

	Mean (SD)		t	p
	Male (n = 536)	Female (n = 383)		
Functional limitation	0.588 (1.2633)	0.963 (1.4824)	− 4.025**	< 0.001
Physical pain	0.853 (1.4872)	1.493 (1.7424)	− 5.837**	< 0.001
Psychological discomfort	0.496 (1.3423)	0.768 (1.4920)	− 2.833**	0.005
Physical disability	0.834 (1.4752)	1.350 (1.6413)	− 4.898**	< 0.001
Psychological disability	0.724 (1.4030)	1.115 (1.4426)	− 4.097**	< 0.001
Social disability	0.556 (1.2769)	0.734 (1.2728)	− 2.084*	0.037
Social handicap	0.588 (1.3041)	0.885 (1.3983)	− 3.269**	0.001
OHIP-14 TOTAL	4.638 (8.5662)	7.308 (8.7510)	− 4.600**	< 0.001

*p < 0.05 (two tailed), **p < 0.01 (two tailed)

## Discussion

This study was the first time to conduct a sex-specific measurement invariance study of the Chinese version of the OHIP-14 scale in a sample of Chinese college students. CFA results showed that the 7-factor model of OHIP-14 fit well in the total sample of Chinese college students, proving that the 7-factor model of OHIP-14 is in the stability among Chinese college students. Also, the results showed that the OHIP-14 had good internal consistency and test- retest reliability. The results of model nesting showed that residual invariance was established, indicating that the scale was an effective measurement tool, and the OHIP-14 scale supported the measurement invariance of gender in Chinese college students. In addition, compared with male college students, female college students scored significantly higher on the OHIP-14 scale than male college students.

Since the Chinese version of OHIP-14 scale was firstly translated in 2006, it has been widely applied in Chinese dental clinical research, such as dental aesthetics [45], oral health of edentulous subjects [46], the children’s oral health and so on [47]. The primary reason for selecting the 7-factor model of OHIP-14 for the measurement invariance analyses was theoretical [48]. As shown in Table 3, the 7-factor model was assumed in this study, its fitting indicators all met the recommended standards [49]. This proved that the questionnaire has good construct validity and was appropriate to the Chinese cultural background. Among the questionnaires completed by the students, as shown in Table 2, the physical pain dimension and the psychological pain dimension had the highest average scores, and the psychological discomfort dimension was the lowest average score. Interestingly, a study by Ashokkumar Thirunavukkarasu et al. on a population of young adults in Saudi Arabia showed that physical pain had the highest OHIP score, followed by psychological discomfort [50]. Besides, an investigation of OHRQoL in 18-year-olds conducted in Hong Kong, China, found that psychological discomfort and mental disability have significant effects [51]. This difference reflected that OHRQoL was culturally dependent.

To test measurement invariance, the following model fit indicators were used: CFI, TLI, RMSEA, and SRMR. Among them, the CFI and TLI values were greater than 0.95, while the RMSEA value less than 0.08 and SRMR values were less than 0.05, indicating that the model fitted better [52]. The data results of this study showed that the 7-factor model had a good fit (CFI = 0.996 > 0.95, TLI = 0.993 > 0.95, SRMR = 0.013 < 0.05, RMSEA= 0.055 < 0.08). At the same time, the measurement invariance test also used three model comparison indicators: Δχ^2^, ΔCFI, and ΔRMSEA values. Since Δχ^2^ is easily affected by the sample size, when the sample size is greater than 300, the *p* value of Δχ^2^ in the study was likely to be significant, so the ΔCFI value was less than or equal to 0.01 and the ΔRMSEA value is less than or equal to 0.015 to judge whether there was a significant difference [43, 44]. Since this study has a large sample size, the group CFA in this study was mainly assessed by the values of ΔCFI and ΔRMSEA. Researchers tested the gender measurement invariance of the OHIP-14 scale by cohort CFA and compared the potential mean differences between the two sample groups. The configural, metric, scalar and residual invariance of the OHIP-14 scale across genders were supported by the survey data. The scalar invariance model of the 7-factor model of OHIP-14 in the sample of Chinese college students of different genders was established, indicating that the Chinese college students of different genders have the same reference point when using OHIP-14, and the comparison between groups is meaningful [53].

Based on the measurement invariance test among genders, we used the T-test to compare scores between men and women. The results of this study found that women generally scored higher overall than men on the OHIP-14 questionnaire. Symptoms of physical pain, functional limitation, and psychological disability were significantly increased in women compared with men. Likewise, women were more likely to experience pain symptoms, according to the Centers for Disease Control and Prevention [54]. The reason would be that females were more sensitive to pain than males [55, 56]. For pain threshold and tolerance to noxious stimuli, a meta-analysis revealed that women were generally more sensitive than men [57]. There was a higher prevalence of pain in females for headache, migraine, temporomandibular pain, and burning mouth pain [58]. In addition, studies reported that patients with TMJ disease diagnosed with chronic orofacial pain experience severe jaw pain, limited jaw function, and psychological impairment, as the disease affects the temporomandibular joint and surrounding muscles [59, 60]. But this does not affect the use of the OHIP-14 in China. The results of this study contributed to the continued validation of OHIP-14 in nonclinical settings, and we believed they provide additional support for the use of the OHIP-14, extending previous studies in both applied and research settings.

There were some limitations to this study that should be addressed in future studies. The research sample was mainly composed of college students in Hunan Province, which may have a certain impact on the representativeness and external validity of the research results. Future research can select a more nationally representative and heterogeneous sample. In addition, we should note that since we examined measurement invariance using the short version of OHIP-49, these results may be specific to this version. We anticipated that measurement invariance would be examined in future studies with all versions of OHIP.

The purpose of this study was to determine the optimal model for the Chinese version of the OHIP-14, to test for gender invariance, and to analyze gender differences. By exploring the structure of the OHIP-14 scale, the scope of application, advantages, and disadvantages of the scale for college students can be clarified. And by exploring the current situation and influencing factors of college students' OHRQoL, targeted intervention strategies can be put forward, and may be useful in scientific research and practical applications. There is a need for further studies to validate our results and potentially extend the OHIP-14 scale to other contexts.

## Conclusions

The result of this study indicated that the Chinese version of the OHIP-14 scale had good reliability and validity in samples. For the existing factor structures reported for the OHIP-14 scale, the 7-factor structure was the most stable and fit the present data best. This present demonstration of residual measurement invariance across genders indicated that the Chinese version of the OHIP-14 could be considered as a reliable, effective, simple, and convenient tool for monitoring pain in patients and for screening for pain symptoms in large populations quickly.

## Supplementary Information

**Additional file 1.** Results from linear regression model with age, gender, educational level, subject as outcome.

## Data Availability

The datasets generated and/or analyzed during the current study are not publicly available due to privacy reasons, but are available from the corresponding author on reasonable request.

## Abbreviations

OHRQoL: The oral health-related quality of life
OHIP-14: The oral health impact profile-14
CFA: Confirmatory factor analysis
TMJ: Temporomandibular joint
GOHAI: The Geriatric Oral Health Assessment Index
OIDP: Oral impacts on daily performances
OHIP: the Oral Health Impact Profile
WLSMV: The robust weighted least squares with mean and variance adjustment estimator
SD: Standard deviation
CFI: The comparative fit index
TLI: The Tucker–Lewis index
SRMR: Standardized root mean square residual
RMSEA: The root means the square error of approximation
ICC: The intraclass correlation coefficient
df: Degree of freedom
CI: Confidence interval
ICC: Intra-class correlation coefficients
