# Supplementary material for: The Chinese version of the Oral Health Impact Profile-14 (OHIP-14) questionnaire among college students: factor structure and measurement invariance across genders
Source: BMC Oral Health. 2022 Sep 17;22:405. doi: 10.1186/s12903-022-02441-6 (PMC9482739; doi:10.1186/s12903-022-02441-6)
Supplement: Supplementary file 1 — Additional file 1. Results from linear regression model with age, gender, educational level, subject as outcome. [file 12903_2022_2441_MOESM1_ESM.docx]

ADDITIONAL FILES 1

1. Supplementary Appendix Table 1. Results from linear regression model with Age, Gender, Educational level, Subject as outcome.

| Variable | Categories | B(95% confdence interval) | Beta | t-vaule | p-vaule |
| --- | --- | --- | --- | --- | --- |
| Age | (continuous variable) | 0.429(0.161,0.697) | 0.121 | 3.142** | 0.002 |
| Gender | Male | -1.636(-2.858,-0.413) | -0.092 | -2.625** | 0.009 |
|  | Female | Reference |  | | |
| Educational level | Bachelor | -0.385(-4.237,3.467) | -0.015 | -0.196 | 0.845 |
|  | Master | 1.872(-2.162,5.905) | 0.069 | 0.911 | 0.363 |
|  | Doctor | Reference |  | | |
| Subject | Arts | 3.165(1.570,4.759) | 0.158 | 3.895** | 0.000 |
|  | Science | 0.956(-0.474,2.385) | 0.051 | 1.312 | 0.190 |
|  | Medical science | 0.885(-0.987,2.785) | 0.036 | 0.928 | 0.354 |
|  | Others | Reference |  | | |

Supplementary Appendix Table 1. Results from linear regression model with Age, Gender, Educational level, Subject as outcome.

Note. B: Unstandardized Coefficients, Beta: Standardized Coefficients .
